# Transient regulation of RNA methylation in human hematopoietic stem cells promotes their homing and engraftment

**DOI:** 10.1038/s41375-022-01761-4

**Published:** 2022-12-02

**Authors:** Xuepeng Wang, Scott Cooper, Hal E. Broxmeyer, Reuben Kapur

**Affiliations:** 1 Department of Microbiology and Immunology, Indiana University School of Medicine, Indianapolis, IN 46202 USA; 2 Herman B Wells Center for Pediatric Research, Department of Pediatrics, Indiana University School of Medicine, Indianapolis, IN 46202 USA

**Keywords:** Haematopoietic stem cells, Cell signalling

## Abstract

Enhancing the efficiency of hematopoietic stem cell (HSC) homing and engraftment is critical for cord blood (CB) hematopoietic cell transplantation (HCT). Recent studies indicate that N^6^-methyladenosine (m^6^A) modulates the expression of mRNAs that are critical for stem cell function by influencing their stability. Here, we demonstrate that inhibition of RNA decay by regulation of RNA methylation, enhances the expression of the homing receptor chemokine C-X-C receptor-4 (CXCR4) in HSCs. We show that YTH N6-methyladenosine RNA binding protein 2 (YTHDF2), a m^6^A reader and FTO α-ketoglutarate dependent dioxygenase (FTO), a m^6^A eraser play an opposite role in this process. Through screening, we identified several FDA-approved compounds that regulate the expression of *YTHDF2* and *FTO* in CB CD34^+^ cells. We show that transient downregulation of *YTHDF2* or activation of *FTO* by using these compounds inhibits *CXCR4* decay in CB HSCs and promotes their homing and engraftment. Our results demonstrate a novel regulation strategy to enhance the function of CB HSCs and provide a translational approach to enhance the clinical efficacy of HCT.

## Introduction

Hematopoietic stem cells (HSCs) are the only cells that give rise to all blood cell types and are critical for successful hematopoietic cell transplantation (HCT) [1]. Cord blood (CB) has been used to treat patients with both malignant and nonmalignant hematological disorders over the last 30 years [2]. However, the limited number of HSCs in a single unit of CB greatly impacts the usage of CB for HCT. Although several efforts have been devoted to ex vivo produce HSCs and enhance CB HCT, development of newer strategies to improve single-CB-unit HCT by enhancing the homing of HSCs, and thus the engraftment efficiency of HSCs is critical [1–5].

When intravenously transplanted HSCs home to the bone marrow (BM) and implant in microenvironmental niches, stromal cell-derived factor-1 (SDF-1)/CXCR4 chemotactic axis plays a major role in this process [6, 7]. By regulating this axis, migration and homing of HSCs from peripheral blood (PB) to BM can be greatly improved. For example, prostaglandin E2 enhances HSC homing by facilitating HSC chemotaxis toward SDF-1 gradients through upregulation of CXCR4 cell surface expression [8]. Mild heat exposure promotes incorporation of CXCR4 into lipid rafts, thus enhancing HSC chemotaxis and engraftment [9]. These reports have demonstrated the use of novel methods to regulate this pathway and encouraged us to further understand the regulatory mechanism(s) needed to develop better and safer methods that can enhance homing and engraftment of HSCs.

N^6^-methyladenosine (m^6^A) is a type of prevalent modification in cellular mRNA and this reversible modification is associated with RNA maturation, translation, localization and decay [10, 11]. Recently, several reports described the function of m^6^A modification in the development of HSCs, however, the role of this modification on HSC homing has never been elucidated [12, 13]. Here, we studied the m^6^A modification on *CXCR4* in HSCs and investigated the function of this modification in HSC homing and engraftment. Our work shows that inhibition of *CXCR4* decay by downregulation of *YTHDF2*, a m^6^A reader or upregulation of *FTO*, a m6A eraser, promotes HSC homing and engraftment. This study elucidates a novel function of m^6^A modification in HSC homing and engraftment and provides a new regulation strategy for future clinical applications.

## Material and methods

### Mice

Immunodeficient 6- to 8-week-old NSG (NOD.Cg-Prkdcscid IL2rgtm1Wjl/SzJ) mice were obtained from the In vivo Therapeutics Core at Indiana University School of Medicine (IUSM). The mice were maintained in the Laboratory Animal Resource Center at IUSM. All animal experiments followed protocols approved by the Institutional Animal Care and Use Committee of IUSM.

### Human CD34^+^ CB cell collection and culture

Umbilical CB units were supplied by Cleveland Cord Blood Bank and CordUse, Orlando, FL, USA. All studies were approved by the institutional review board of the IUSM. Mononuclear cells were isolated by density gradient centrifugation and CD34^+^ cells were isolated by immunomagnetic selection kit (Miltenyi Biotec, 130-046-702). The purity of human CD34^+^ CB cells was over 90% and the cells were cultured in StemSpanII serum-free medium (STEMCELL Technologies, 09650) supplemented with 100 ng/ml stem cell factor (SCF), 100 ng/ml FMS-like tyrosine kinase 3 ligand (Flt3L) and 100 ng/ml thrombopoietin (TPO). For molecular compound treatment, all reagents were ordered from Cayman Chemical (Ann Arbor, MI, USA). Briefly, molecular compounds were added in the initiation of human CB CD34^+^ cells culture, the time and concentration of each molecular compound for cell treatment was shown in figure legends.

### RNA extraction and real-time PCR

After cells were harvested by FACS, RNA was extracted by a RNeasy Mini Kit following manufacturer’s protocol (Qiagen, 74106). Total RNA was reverse-transcribed by use of Superscript III kit (Thermo Fisher, 18080093). Quantitative real-time PCR reactions were performed by SYBR Green PCR Master Mix (Thermo Fisher, Florence, KY, USA) and an Agilent Mx3000P QPCR System. Expression of housekeeping gene *GAPDH* was used as an internal control. Data are shown as relative mRNA level normalized to levels in vehicle control, set to 1. The sequence of the primers are listed in Supplementary Table 1.

### siRNA transfection

siRNA oligos were synthesized by Genewiz. Non-targeting siRNA was used as the control. The siRNA duplexes sequences used to target YTHDF2 is 5′-TTGGCTATTGGGAACGTCCTT-3′. For electroporation, human CD34^+^ cells were resuspended at 1 × 10^6^ cell/ml in StemSpanII serum-free medium and thereafter electroporated using the CD34^+^ Cell Nucleofector Kit and Nucleofector II device from Amaxa. Control, si-YTHDF2 or a fluorescently labeled, non-targeting siRNA at a final concentration of 2 μM was added in a volume of 10 μl Nucleofector Solution. Briefly, 200 ul human CD34^+^ cells were pelleted for 5 min at 400 × *g* and resuspended in 10 μl the prepared Nucleofector Solution. Cell suspension was transferred into a cuvette and electroporated with program U-008 for human CD34^+^ cells. After electroporation, 500 μl pre-warmed StemSpanII serum-free medium was added to the cuvette and then the suspension was transferred into a 24-well plate. In vitro or in vivo assays were performed after 24 h. Transfection efficiencies were analyzed by calculating the percentage of fluorescent cells regarding the total cell.

### RNA immunoprecipitation

RNA immunoprecipitation was performed as previously described [14]. Briefly, 0.5–1 × 10^6^ collected human CD34^+^ cells were lysed in buffer A (20 mM Tris-Cl, pH 8.0; 100 mM NaCl, 1 mM EDTA, 0.5% NP-40, protease and RNase inhibitors) for 20 min at 4 °C. After centrifugation for 5 min, 1/10th volume of cell lysate was saved as input. Anti-YTHDF2 (Abcam, ab220163) was prebound to the protein A/G beads and then reacted with the rest of the cell lysate overnight at 4 °C. After elution from the beads, the RNA samples were precipitated with ethanol and dissolved in RNase-free water. RNAs were further purified with RNA Clean and Concentrator-5 (Zymo, R1013). The relative CXCR4 enrichment was determined by calculating the Ct values of the RIP sample relative to the input sample. Expression of non-m6a housekeeping gene ACTB was used as an internal control. Data are shown as relative binding level normalized to levels in sh-Control group, set to 1.

### M6A-RNA immunoprecipitation

MeRIP analysis was used as previously described [15]. Briefly, the total RNAs were extracted, and then RNA concentration was adjusted to 1 μg/μl. RNA was fragmented into ∼100 nt size and these RNA were immunoprecipitated with the anti-m6A antibody according to the standard protocol of the Magna MeRIP m6A Kit (Merck Millipore, 17-10499). M6A enrichment was determined by qPCR analysis.

### Vector construction and virus production

*FTO* and *ALKBH5* cDNA were cloned from CB CD34^+^ cell cDNA library. Briefly, fresh CB CD34^+^ cells were harvested and total RNA extracted. The total RNA was reverse-transcribed by use of Superscript III kit. The cDNA was then ligated into an overexpression plasmid by use of In-Fusion HD Cloning Plus kit (Takara, 638920). For shRNA, microRNA and anti-microRNA vector construction, single-strand oligos were synthesized by Genewiz. After annealing, double-strand oligos were ligated into the shRNA expression plasmid by T4 ligase (NEB, M2622L). Well-constructed vectors were sent to Genewiz (South Plainfield, NJ, USA) for sequencing.

### Lentivirus transduction of CB CD34^+^ cells

Lentiviruses were concentrated by 30% percutaneous endoscopic gastrostomy 8000 (PEG8000, Sigma, 1546605). Before transduction, freshly isolated CB CD34+ cells were cultured in stemspanII medium with SCF, Flt3L and TPO for 6 h, 20 μm CsH was added to the medium and cells were cultured for 16 h. Lentivirus was added in the same medium at a MOI of 50–200 and incubated for 8 h, a step which was repeated three times. GFP positive cells were identified as lentivirus transduced cells.

### Flow cytometry

Fresh or cultured CB cells were sorted for different phenotypes by using the following antibodies. Antibodies were used to detect CD34 (BV786, BD Biosciences, 744741), CD34 (FITC, BD Biosciences, 340668), CD45RA (Taxe-red, BD Biosciences, 562298), CD90 (BV421, BD Biosciences, 562556), CD49f (cy5.5, BD Biosciences, 562495), CD38 (PE, BD Biosciences, 560981). CD3 (BV421, BD Biosciences, 562427), CD33 (PE, BD Biosciences, 555450), CD19 (PE, BD Biosciences, 555413), CXCR4 (APC, BD Biosciences, 555976) and CD45 (APC, BD Biosciences, 555485). Gating strategy of phenotypic HSCs were shown in Supplementary Fig. 2B.

### Chemotaxis assay

Chemotaxis assays were performed in Costar 24-well transwell plates with 6.5 mm diameter inserts with 5.0 μm pores (Corning, NY, USA). Briefly, 650 μl of IMDM medium (37 °C) that contained 0.5% bovine serum albumin (Sigma-Aldrich) and SDF-1 (0, 50 ng/ml) was added to the bottom well. Cells were suspended at 1 × 10^5^ cells/100 μl in IMDM medium and loaded to the upper chamber of the transwell. Transwell plates were placed in a 37 °C incubator with 95% humidity and 5% CO_2_ for 4 h. Percent migration was measured using flow cytometry with number of cells in the bottom chamber divided by number of cells placed in the upper chamber. To calculate the percent migration of CB HSCs, phenotypic HSCs was determined by surface staining and flow cytometry analysis. Human CB HSC chemotaxis was calculated as number of HSCs in the bottom chamber divided by number of HSCs loaded in the upper chamber. For AMD3100 administration, cells were treated with 5 μg/ml AMD3100 (239820, Sigma- Aldrich) for 30 min right before the chemotaxis assay.

### Homing assay

Homing of human CB CD34^+^ cells was evaluated in NSG mice. Molecular compounds treated or siRNA transfected human CB CD34+ cells (500,000 for each mouse) were intravenously injected into sublethally irradiated (350 cGy) NSG mice. After 24 h, these recipient mice were sacrificed, BM cells from each mouse were collected. Cells were stained with anti-human CD45 antibody, then resuspended in 1% formaldehyde buffer. Flow cytometry analysis was performed to determine the percentage of human CD45^+^ cells. BM cells from non-transplanted NSG mice served as negative controls.

### Colony-forming unit (CFU) assay

GFP^+^, CD34^+^ cells harvested after cell sorting by FACS were plated in semi-solid methylcellulose culture medium in presence of 30% Fetal bovine serum (GE Healthcare, HyClone, SH30071.03), 2 mM l-glutamine (Lonza, 17-605E), 100 μM β-mercaptoethanol (Sigma, M6250), 1 U/ml erythropoietin (EPO) (R&D Systems, 287-TC-500), 50 ng/ml SCF (R&D Systems, 7466-SC-010/CF), 10 ng/ml IL-3 (R&D Systems, 203-IL-050/CF) and 10 ng/ml GM-CSF (R&D Systems, 7954-GM-010) and were cultured at lower (5%) O_2_ and at 5% CO_2_ in a humidified incubator. Number of CFU-GM- and CFU-GEMM-colonies were scored with an inverted microscope 14 days after culture in semi-solid medium. This culture medium allows detection of CFU-GM- and CFU-GEMM-colonies, but not BFU-E colonies.

### Limiting dilution analysis (LDA)

Frequency of human SCID repopulating cells (SRCs) was determined by LDA as reported before. Increasing doses of GFP overexpressing CD34^+^ cells (2500, 5000 or 10,000 cells) were intravenously injected into sublethally irradiated NSG recipient mice (350 cGy; 137Cs source, single dose). Four months after transplantation, the percentage of GFP^+^ human CD45^+^ cell chimerism was analyzed by immunostaining and flow cytometry. For long-term engraftment assays that assess the self-renewal capacity of HSC, 3 × 10^6^ BM cells from primary recipients of the 10,000-cell group were intravenously transplanted into secondary sublethally irradiated NSG recipient mice.

### mRNA stability assay

Each sample was harvested at 4 and 6 h after treatment with actinomycin D (2 μM). Total RNA was isolated with the RNeasy plus mini kit (QIAGEN). The HPRT1 housekeeping gene was used as a loading control.

### Statistical analysis

Statistical analysis was performed by use of Microsoft Excel and GraphPad Prism (GraphPad Software, San Diego, CA, USA). Data are shown as mean ± s.e.m. (as indicated in the figure legends). One-way ANOVA was used to compare differences in means between more than two groups, as indicated.

## Results

### Downregulation of *YTHDF2* or upregulation of *FTO* inhibits *CXCR4* decay

**Fig. 1:**
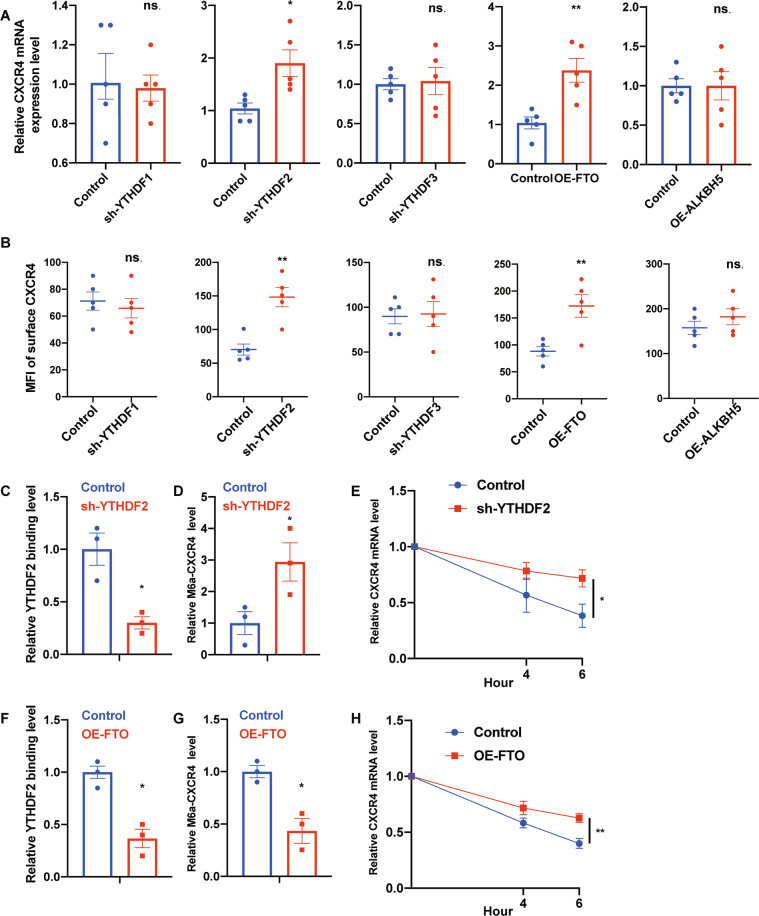
Downregulation of *YTHDF2* or upregulation of *FTO* inhibits CXCR4 decay. **A** Relative *CXCR4* mRNA expression in human CB CD34^+^ cells after *YTHDF1*, *YTHDF2* and *YTHDF3* downregulation or *FTO* and *ALKBH5* overexpression. Data pooled from three independent experiments are shown (*n* = 5 cultures per group). **B** Quantification of mean fluorescence intensity (MFI) of surface CXCR4 expression on human CB CD34^+^ cells. Data pooled from three independent experiments are shown (*n* = 5 cultures per group). **C** YTHDF2 binding level of *CXCR4*, verified by YTHDF2 RIP combined with RT-qPCR. Data pooled from two independent experiments are shown (*n* = 3 cultures per group). **D** m6A enrichment on *CXCR4*, verified by m6A IP combined with RT-qPCR. Data pooled from two independent experiments are shown (*n* = 3 cultures per group). **E** Representative mRNA profile of CXCR4 after actinomycin D treatment in control and sh-YTHDF2 transduced human CB CD34^+^ cells. Data pooled from three independent experiments are shown (*n* = 5 cultures per group). **F** YTHDF2 binding level of *CXCR4* in *FTO* overexpressing human CB CD34^+^ cells, verified by YTHDF2 RIP combined with RT-qPCR. Data pooled from two independent experiments are shown (*n* = 3 cultures per group). **G** m6A enrichment on CXCR4, verified by m6A IP combined with RT-qPCR. Data pooled from two independent experiments are shown (*n* = 3 cultures per group). **H** Representative mRNA profile of *CXCR4* after actinomycin D treatment in control and *FTO* overexpressing human CB CD34^+^ cells. Data pooled from three independent experiments are shown (*n* = 5 cultures per group). Data shown as mean ± s.e.m. *p < 0.05; **p < 0.01; ***p < 0.001 by one-way ANOVA.

*CXCR4* is one of the most important mediators in regulating the homing of HSCs. Therefore, we focused on this gene and hypothesized that the epigenetic regulation on mRNA contributes to the expression of *CXCR4* and HSC homing. To this end, previous published m^6^A-seq data has shown that *CXCR4* is labeled by m^6^A in human CB CD34^+^ cells (Fig. S1A) [12]. Based on this, we conducted loss-and-gain of function experiments to explore the regulators that modulate RNA methylation of *CXCR4*. Five m^6^A regulators were measured in these experiments. As shown previously, *YTHDF* functions to mediate the decay of m^6^A-mRNAs [16, 17]. By using a lentivirus based knockdown strategy, 3 members of the YTH N6-methyladenosine RNA binding protein (*YTHDF)* family (*YTHDF1*, *YTHDF2* and *YTHDF3*), were downregulated in CB CD34+ cells (Fig. S1B). We found that only downregulation of *YTHDF2* upregulated *CXCR4* expression at RNA level as well at the protein level (Figs. 1A, B and S1D). These results suggest that *YTHDF2* plays a key role in regulating the decay of *CXCR4* mRNA in human CD34^+^ cells. In contrast, overexpression of two different m^6^A demethylases, *FTO* and alkB homolog 5 (*ALKBH5)* by using lentivirus, induced a very different phenotype (Fig. S1C) [15, 18, 19]. We show that, overexpression of *FTO* demethylase but not *ALKBH5*, promotes CXCR4 expression (Figs. 1A, B and S1D). These results suggest that removal of the m^6^A tag from the *CXCR4* mRNA attenuates its decay.

In a protein binding assay, we found that when *YTHDF2* was knocked down using a shRNA in human CD34^+^ cells, the binding levels of YTHDF2 and *CXCR4* were greatly decreased (Fig. 1C) and the relative m^6^A labeled *CXCR4* levels were dramatically increased (Fig. 1D). In a mRNA stability assay, after actinomycin D treatment of human CB CD34^+^ cells for 6 h, the relative *CXCR4* mRNA levels were significantly upregulated when *YTHDF2* was downregulated by shRNA (Fig. 1E). Actinomycin D is a transcription inhibitor and widely used in mRNA stability assays. Actinomycin D can form a very stable complex with DNA and thus inhibiting the DNA-dependent RNA polymerase activity, allowing the assessment of mRNA stability by measuring their abundance following transcription inhibition [20]. Our results suggest that YTHDF2 can bind m^6^A labeled *CXCR4* in human CB CD34^+^ cells resulting in its decay. Downregulation of YTHDF2 induced lower binding level between YTHDF2 and *CXCR4*. Eventually, higher expression level of CXCR4 was detected in YTHDF2 downregulated group. When we overexpressed *FTO* in human CB CD34^+^ cells, the level of binding between YTHDF2 and *CXCR4* was reduced (Fig. 1F), as well as the relative m^6^A labeled *CXCR4* level (Fig. 1G). When we treated human CB CD34^+^ cells with actinomycin D for 6 h, the *CXCR4* mRNA levels were significantly increased (Fig. 1H). These results suggest that *FTO* functions as an “eraser” to remove m^6^A on *CXCR4*, which inhibits *CXCR4* decay by decreasing the binding of YTHDF2 to *CXCR4*.

### Transient knockdown of *YTHDF2* by siRNA promotes human HSC migration and homing

**Fig. 2:**
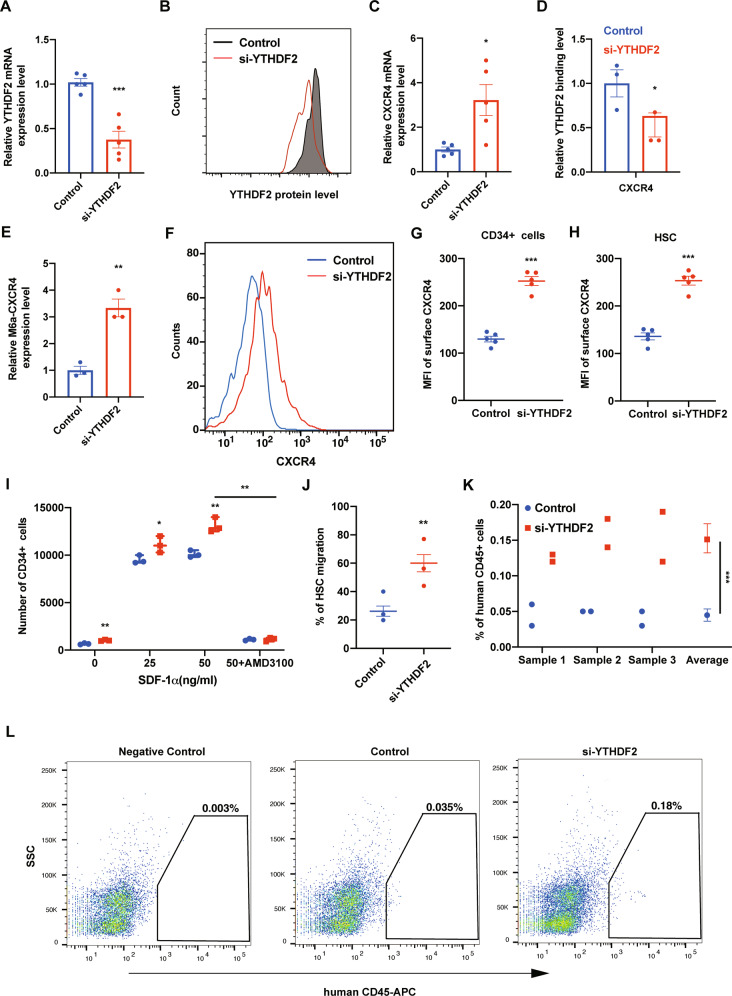
Transient knockdown of *YTHDF2* by siRNA promotes human HSC migration and homing. **A** Relative *YTHDF2* expression level in human CB CD34^+^ cells after *YTHDF2* knockdown. Data pooled from three independent experiments are shown (*n* = 5 cultures per group). **B** Histogram showing intracellular flow validation of *YTHDF2* knockdown in human CB CD34^+^ cells. Representative images from two independent experiments are show. **C** Relative CXCR4 protein level in human CB CD34^+^ cells after *YTHDF2* knockdown. Data pooled from three independent experiments are shown (*n* = 5 cultures per group). **D** YTHDF2 binding level of *CXCR4* in Control or si-YTHDF2 transfected human CB CD34^+^ cells, verified by YTHDF2 RIP combined with RT-qPCR. Data pooled from two independent experiments are shown (*n* = 3 cultures per group). **E** m6A enrichment on *CXCR4*, verified by m6A IP combined with RT-qPCR. Data pooled from two independent experiments are shown (*n* = 3 cultures per group). **F** Histogram of surface CXCR4 protein level of Control or si-YTHDF2 transfected human CB CD34^+^ cells. Representative histogram from five independent experiments is shown. **G** Quantification of mean fluorescence intensity (MFI) of surface CXCR4 protein level of Control or si-YTHDF2 transfected human CB CD34^+^ cells. Data pooled from five independent experiments are shown (*n* = 5 cultures per group). **H** Quantification of mean fluorescence intensity (MFI) of surface CXCR4 of Control or si-YTHDF2 transfected human HSCs (CD34^+^ CD38^−^ CD45RA^−^ CD90^+^ CD49f^+^). Data pooled from five independent experiments are shown (*n* = 5 cultures per group). **I** Chemotaxis of human CB CD34+ cells toward human recombinant SDF-1, as quantified by flow cytometry. Data pooled from three independent experiments are shown (each dot represents an independent chemotaxis). **J** Migration of human phenotypic HSCs in CB CD34+ cells toward human recombinant SDF-1 (50 ng/ml), as quantified by flow cytometry. The migration percentage of HSCs was calculated by analyzing the HSC (CD34^+^ CD38^−^ CD45RA^−^ CD90^+^ CD49f^+^) frequency using flow cytometry. Data pooled from five independent experiments are shown (each dot represents an independent chemotaxis). **K** The percentage of human CD45^+^ cells in the BM of NSG mice 24 h after transplantation with 500,000 CB CD34+ cells that had been transfected with Control or si-YTHDF2. CD34^+^ cells from three CB samples (CB 1–CB 3) were tested (*n* = 5 mice per group). **L** Representative flow cytometric analysis of human cells in the BM of NSG mice, 24 h after transplantation. Left is from a mouse without transplantation (negative control), center and right are from mice transplanted with human CB CD34+ cells transfected with Control or si-YTHDF2. Human engraftment was assessed as the percentage of human CD45^+^ cells. Data shown as mean ± s.e.m. *p < 0.05; **p < 0.01; ***p < 0.001 by one-way ANOVA.

As homing is a short-term process and in theory, transfection of siRNA into human CB CD34^+^ cells can transiently induce and maintain downregulation of *YTHDF2*, we transfected human CB CD34^+^ cells with siRNA by electroporation. siRNA efficiently downregulated YTHDF2 expression at the RNA and at the protein level in human CB CD34^+^ cells (Figs. 2A, B and S2A). When YTHDF2 was knockdown by siRNA, the RNA level of *CXCR4* was significantly upregulated (Fig. 2C). The binding level of YTHDF2 and *CXCR4* was significantly decreased (Fig. 2D) and the expression level m^6^A labeled *CXCR4* was greatly increased (Fig. 2E). These results suggest that knockdown of *YTHDF2* by siRNA is feasible in human CB CD34+ cells. Furthermore, flow cytometry analysis of knockdown cells revealed that CXCR4 protein level was notably upregulated in human CB CD34^+^ cells, as well as in the rigorously defined population of HSCs (CD34^+^ CD38^−^ CD45RA^−^ CD49f^+^ CD90^+^) (Figs. 2F–H and S2B, C). The effect of si-YTHDF2 on HSC chemotaxis was evaluated in an in vitro transwell migration assay. siRNA transfected CB CD34^+^ cells showed significant migration to SDF-1 (Fig. 2I). Enhanced migration to SDF-1 by siRNA transfection was also observed in the HSC population (Fig. 2J). Chemotaxis of human CB CD34^+^ cells to SDF-1 was blocked by CXCR4 antagonist AMD3100, suggesting that the enhanced migration was mediated through CXCR4 (Fig. 2I). To further investigate the role of *YTHDF2* knockdown by siRNA on homing, siRNA transfected human CB CD34^+^ cells were transplanted into NSG mice. After 24 h, the human CD45^+^ cells that homed to the BM were analyzed by flow cytometry. siRNA transfection enhanced human CB CD34^+^ cell homing in NSG mice (Fig. 2K, L). These results suggest that transient knockdown of *YTHDF2* by siRNA promotes human HSC migration and homing.

### Transient downregulation of *YTHDF2* by siRNA enhanced human CB CD34^+^ cell engraftment

**Fig. 3:**
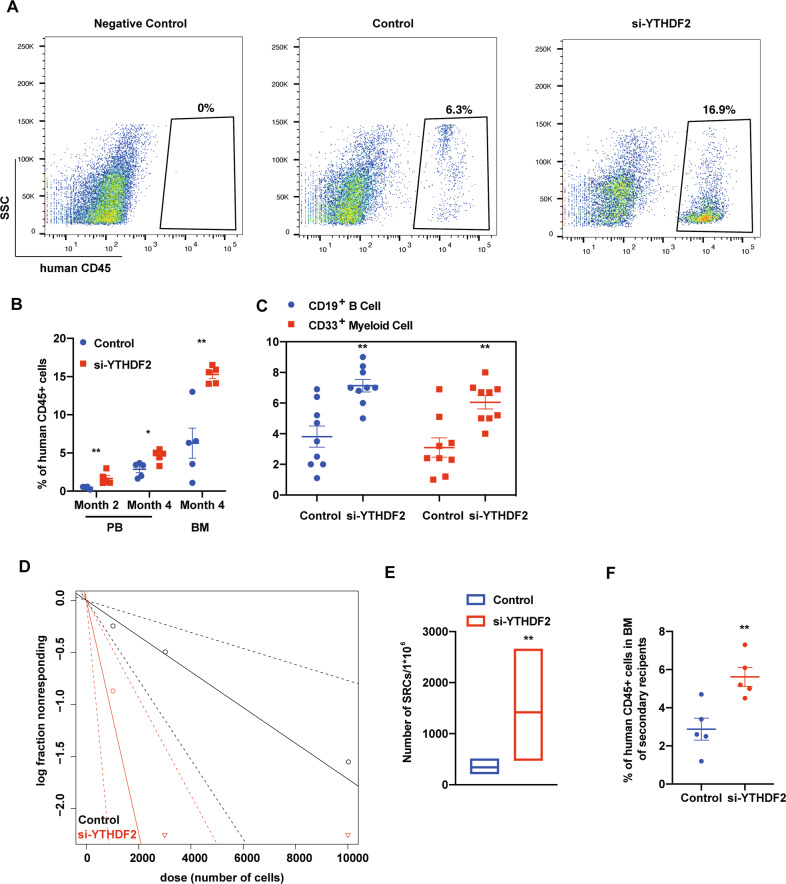
Transient downregulation of *YTHDF2* by siRNA enhanced human CB CD34^+^ cell engraftment. **A** Representative flow cytometric analysis of human engraftment in the BM of NSG mice, 4 months after transplantation. Left is from a mouse without transplantation (negative control), center and right are from mice transplanted with human CB CD34+ cells transfected with Control or si-YTHDF2 Human engraftment was assessed as the percentage of human CD45^+^ cells. **B** The percentage of human CD45+ cells in the PB and BM of NSG mice at the indicated time points after transplantation with 10,000 CB CD34^+^ cells that had been transfected with Control or si-YTHDF2 (*n* = 5 in each group). **C** The percentage of human CD33^+^ myeloid cell and CD19^+^ B-cell in BM was determined 4 month after transplantation (*n* = 9 or 10 mice for each group). **D** Poisson statistical analysis. *n* = 30 mice in total. Shapes represent the percentage of negative mice for each dose of cells. Solid lines indicate the best-fit linear model for each data set. Dotted lines represent 95% confidence intervals. **E** HSC frequencies (line in the box) and 95% confidence intervals (box) presented as the number of SRCs in 1 × 10^6^ CD34^+^ cells. **F** Human CD45^+^ cell chimerism in the BM of secondary recipient NSG mice at 4 months, which had been transplanted with 5 × 10^6^ BM cells from primary recipient NSG mice. (*n* = 5 in each group). Data shown as mean ± s.e.m. *p < 0.05; **p < 0.01; ***p < 0.001 by one-way ANOVA.

We next evaluated the long-term engraftment of siRNA transfected human CB CD34^+^ cells by limiting dilution assay (LDA). LDA results indicate that siRNA transfected human CB CD34^+^ cells display enhanced engraftment relative to the recipients of control group both in the PB at 2 and 4 months, respectively and in the BM at 4 months post transplantation (Fig. 3A, B). Human myeloid and B-cell chimerism were also significantly increased (Figs. 3C and S3A). Poisson distribution analysis revealed an SRC frequency of 1/2439 for the control group and 1/667 for the siRNA transfected group. This reflected respectively 410 and 1478 SRCs in 1 × 10^6^ cells from control and siRNA transfected group, resulting in a ~4-fold increase in the number of functionally detectable SRCs compared with the control group (Fig. 3D, E and Table S2). Transplanting BM cells from the siRNA transfected group into secondary NSG mice, also resulted in the enhanced engraftment (Fig. 3F). These data suggest that the long-term self-renewal capability of human CB HSCs was enhanced by transient siRNA mediated knockdown of *YTHDF2*.

### A small-scale screen identified several compounds that functionally downregulate *YTHDF2* and enhance human CB CD34^+^ cell migration and homing

**Fig. 4:**
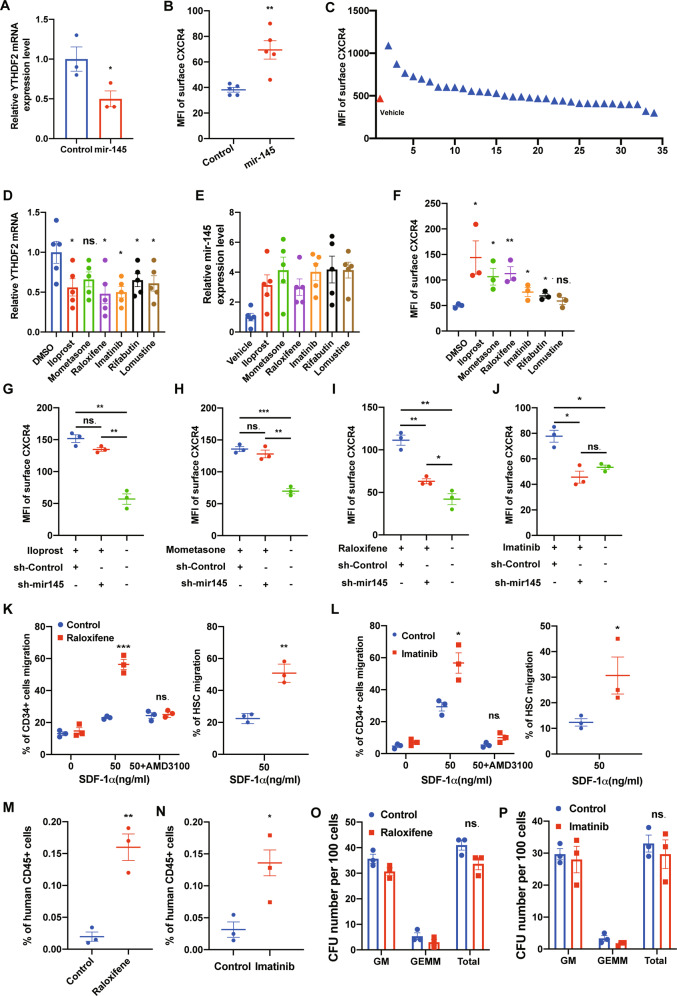
A small-scale screen identified several compounds that functionally downregulate *YTHDF2* and enhance human CB CD34^+^ cell migration and homing. **A** Relative YTHDF2 expression level in mir-145 transduced human CB CD34^+^ cells. Data pooled from three independent experiments are shown (*n* = 3 cultures per group). **B** Quantification of mean fluorescence intensity (MFI) of surface CXCR4 of human CB CD34^+^ cells. Data pooled from three independent experiments are shown (*n* = 5 cultures per group). **C** Mean fluorescence intensity (MFI) of cell surface CXCR4 protein level on human CB CD34^+^ cells after treatment for 20 h with compounds. The concentration of each compound used in this screen was 10 μM unless otherwise stated. The *y* axis represents relative fluorescence units (RFU) calculated by FlowJo. **D** Relative *YTHDF2* expression level in representative compounds treated human CB CD34^+^ cells. Data pooled from two independent experiments are shown (*n* = 5 cultures per group). **E** Relative mir-145 expression level in representative compounds treated human CB CD34^+^ cells. Data pooled from three independent experiments are shown (*n* = 5 cultures per group). **F** Mean fluorescence intensity (MFI) of cell surface CXCR4 protein level on human CB CD34^+^ cells after treatment for 20 h with representative compounds. Data pooled from three independent experiments are shown (*n* = 3 cultures per group) **G**–**J** Mean fluorescence intensity (MFI) of cell surface CXCR4 on representative compounds treated human CB CD34^+^ cells combine with Control or sh-mir-145. Data pooled from three independent experiments are shown (*n* = 3 cultures per group). **K** Raloxifene (40 μM, 20 h) treated human CB CD34^+^ cells (left) and phenotypic HSC (right) migration toward human recombinant SDF-1, as quantified by flow cytometry. Data pooled from three independent experiments are shown. (*n* = 3 cultures per group). **L** Imatinib (10 μM, 20 h) treated human CB CD34^+^ cells (left) and phenotypic HSC (right) migration toward human recombinant SDF-1, as quantified by flow cytometry. Data pooled from three independent experiments are shown. (*n* = 3 cultures per group). **M** The percentage of human CD45^+^ cells in the BM of NSG mice 24 h after transplantation with 500,000 CB CD34+ cells that had been treated with DMSO or Raloxifene (40 μM, 20 h) (*n* = 3 in each group). **N** The percentage of human CD45^+^ cells in the BM of NSG mice 24 h after transplantation with 500,000 CB CD34+ cells that had been treated with DMSO or Imatinib (10 μM, 20 h) (*n* = 3 in each group). **O** Colony output of DMSO or Raloxifene (40 μM, 20 h) treated CB CD34^+^ cells. Data pooled from two independent experiments are shown. (*n* = 3 cultures per group). **P** Colony output of DMSO or Imatinib (10 μM, 20 h) treated CB CD34^+^ cells. Data pooled from two independent experiments are shown. (*n* = 3 cultures per group). Data shown as mean ± s.e.m. *p < 0.05; **p < 0.01; ***p < 0.001 by one-way ANOVA.

In previous reports, *mir-145* was identified as a repressor of *YTHDF2* by targeting the 3’-untranslated mRNA region of *YTHDF2* [21, 22]. Overexpression of *mir-145* in human CB CD34^+^ cells resulted in notable downregulation of *YTHDF2* (Fig. 4A). Using flow cytometry, we observed an upregulation of CXCR4 protein level in human HSCs overexpressing *miR-145* (Fig. 4B). These results demonstrate that *mir-145* can regulate *YTHDF2* expression and eventually the expression of CXCR4 in human HSCs. By mining a public database, Psmir, we identified 33 candidate compounds that could induce *mir-145* expression [23]. We treated human CB CD34^+^ cells with these compounds for 20 h and assessed the expression of CXCR4, *YTHDF2* and *mir-145* (Fig. 4C–E). We found Iloprost, Mometasone, Raloxifene and Imatinib strongly upregulated mir-145 and CXCR4 expression and downregulated *YTHDF2* expression (Fig. 4D–F). Iloprost is a synthetic analog of prostacyclin [24]. It’s well known that prostaglandins and their receptors have important function in regulating HSC homing [8]. Mometasone, a type of glucocorticoid, is known to boost HSC homing [25]. When human CB CD34^+^ cells were treated with these compounds and sh-mir-145, we found that inhibition of *mir-145* did not affect the function of Iloprost and Mometasone on *CXCR4* activation (Fig. 4G, H). In contrast, the impact of Raloxifene and Imatinib on CXCR4 expression in human CB CD34^+^ cells were significantly blocked by inhibition of *mir-145* (Fig. 4I, J). These results suggest that Raloxifene and Imatinib upregulate CXCR4 expression mainly via activation of *mir-145* expression. Iloprost and Mometasone likely regulate CXCR4 expression through more complicated mechanisms as previously reported [8, 25]. The optimal concentration and time of treatment to induce maximal CXCR4 expression by Raloxifene and Imatinib in human CB CD34^+^ cells were evaluated in detail (Fig. S4A–D). We next assessed whether Raloxifene, a FDA-approved drug to treat osteoporosis and Imatinib treatment can promote chemotaxis of human CB HSCs toward SDF-1. Raloxifene and Imatinib treatment significantly enhanced chemotaxis of human CB CD34^+^ cells toward SDF-1, which could be fully blocked by CXCR4 antagonist AMD3100 (Fig. 4K, L). Enhanced migration to SDF-1 by Raloxifene and Imatinib was also observed in phenotypic HSC population (Fig. 4K, L). We next tested whether Raloxifene and Imatinib treatment of CB CD34+ cells can enhance the homing of these cells in vivo. Human CB CD34^+^ cells were treated with the compounds for 20 h and then transplanted into NSG mice. We found that both Raloxifene and Imatinib treatment dramatically increased the homing efficiency of human CB CD34^+^ cells (Fig. 4M, N). In colony-forming assay, no significant difference was observed in Imatinib and Raloxifene treated cells when compared with the control group, respectively (Fig. 4O, P). Thus, imatinib and Raloxifene treatment promotes human CB CD34^+^ cell migration and homing through downregulation of *YTHDF2*.

### Raloxifene and Imatinib treatment promotes human CB CD34^+^ cell engraftment

**Fig. 5:**
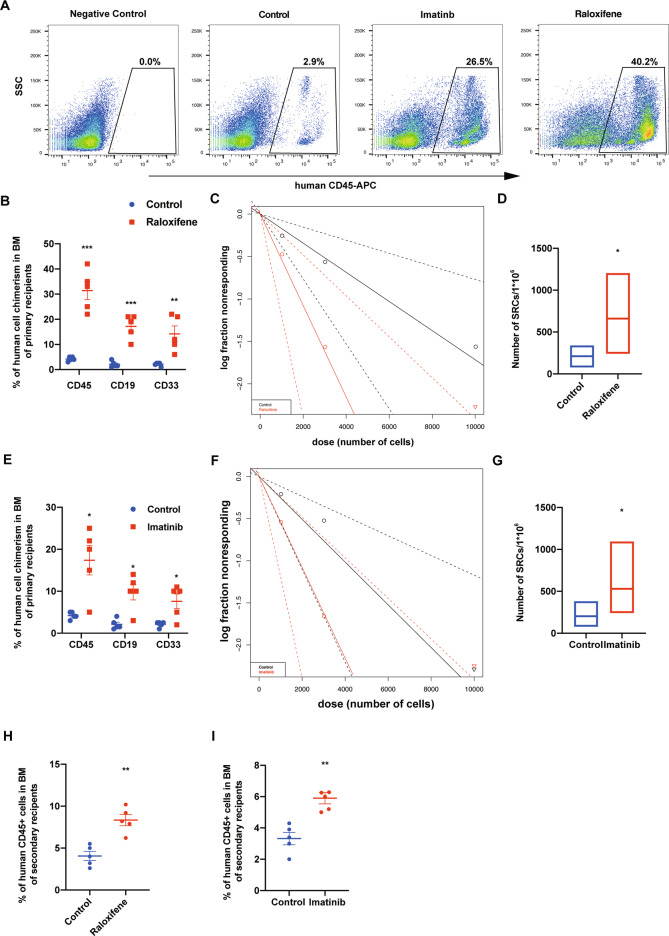
Raloxifene and Imatinib treatment promotes human CB CD34^+^ cells engraftment. **A** Representative flow cytometric analysis of human engraftment in the BM of NSG mice, 4 months after transplantation. Left is from a mouse without transplantation (negative control), center and right are from mice transplanted with human CB CD34^+^ cells treated with DMSO control, Imatinb (10 μM) or Raloxifene (40 μM) for 20 h. Human engraftment was assessed as the percentage of human CD45^+^ cells. **B** The percentage of human CD45^+^ cells, B-cell (CD19^+^), and myeloid cell (CD33^+^) chimerism in the BM of NSG mice after transplantation with 10,000 CB CD34+ cells that had been treated with DMSO or Raloxifene. (*n* = 5 mice per group). **C**, **D** The frequency of human SRCs in CB CD34+ cells treated with DMSO or Raloxifene. **E** The percentage of human CD45^+^ cells, B-cell (CD19^+^), and myeloid cell (CD33^+^) chimerism in the BM of NSG mice after transplantation with 10,000 CB CD34+ cells that had been treated with DMSO or Imatinib. (*n* = 5 mice per group). **F**, **G** The frequency of human SRCs in CB CD34+ cells treated with DMSO or Imatinib. **H**, **I** Human CD45^+^ cell chimerism in the BM of secondary recipient NSG mice at 4 months, which had been transplanted with 5 × 10^6^ BM cells from primary recipient NSG mice (*n* = 5 in each group). Data shown as mean ± s.e.m. *p < 0.05; **p < 0.01; ***p < 0.001 by one-way ANOVA.

Long-term engraftment of human CB CD34^+^ cells was assessed in recipients transplanted with vehicle, Imatinib and Raloxifene treated CD34^+^ cells. Imatinib and Raloxifene treated human CB CD34^+^ cells showed significantly elevated engraftment in NSG mice compared with that of the vehicle control-treated group (Fig. 5A). Human myeloid and B-cell chimerism were also increased (Fig. 5B, E). The frequency of SRCs was calculated by LDA. A Poisson distribution analysis revealed an SRC frequency of 1/4761 for vehicle-treated CB CD34^+^ cells and an enhanced SRC frequency of 1/1818 and 1/1406 for Raloxifene and Imatinib treated human CB CD34^+^ cells, respectively. Enhanced engraftment of Imatinib and Raloxifene treated human CB CD34^+^ cells was also apparent in transplanted secondary recipients as compared to vehicle-treated cells (Fig. 5H, I). Thus, treatment of human CB CD34^+^ cells with Raloxifene and Imatinib enhances their long-term engraftment.

### Angiotensin II promotes human CD34^+^ cell migration and homing through activation of *FTO*

**Fig. 6:**
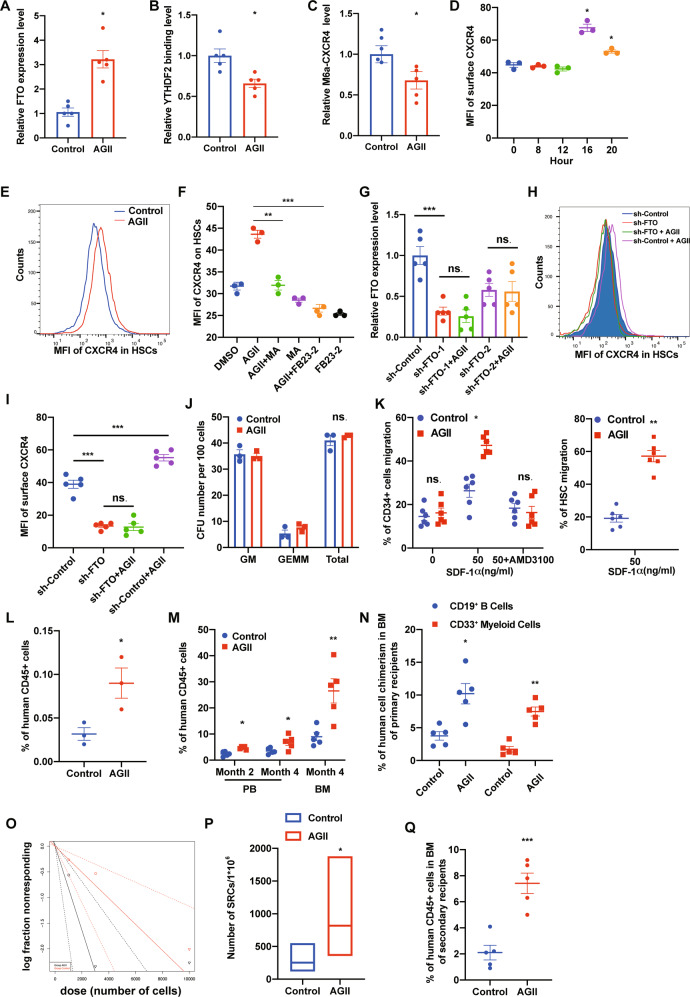
Angiotensin II promotes human CD34^+^ cell migration, homing and engraftment through activation of *FTO*. **A** Relative FTO expression level in vehicle (PBS) or Angiotensin II (AGII, 4 μM) treated human CB CD34^+^ cells. Data pooled from three independent experiments are shown (*n* = 5 cultures per group). **B** YTHDF2 binding level of *CXCR4* in vehicle or AGII treated human CB CD34^+^ cells, verified by YTHDF2 RIP combined with RT-qPCR. Data pooled from two independent experiments are shown (*n* = 5 cultures per group). **C** m6A enrichment on CXCR4 in vehicle or AGII treated human CB CD34^+^ cells, verified by m6A IP combined with RT-qPCR. Data pooled from two independent experiments are shown (*n* = 5 cultures per group). **D** Quantification of mean fluorescence intensity (MFI) of surface CXCR4 protein level of human CB CD34^+^ cells treated with vehicle or AGII. Data pooled from three independent experiments are shown. Data pooled from three independent experiments are shown (*n* = 3 cultures per group). **E** Histogram of surface CXCR4 expression of human CB CD34^+^ cells treated with vehicle or AGII. Representative histogram from three independent experiments is shown. **F** Quantification of mean fluorescence intensity (MFI) of surface CXCR4 of human CB CD34^+^ cells treated with representative compounds. Data pooled from three independent experiments are shown (*n* = 3 cultures per group). **G** Relative *FTO* expression level in representative group of human CB CD34^+^ cells (*n* = 5 cultures per group). **H** Histogram of surface CXCR4 expression in representative group of human CB CD34^+^ cells. **I** Quantification of mean fluorescence intensity (MFI) of surface CXCR4 of human CB CD34^+^ cells treated with representative compounds and shRNA. Data pooled from five independent experiments are shown. (*n* = 5 cultures per group). **J** Colony output of vehicle or AGII treated CB CD34^+^ cells. Data pooled from two independent experiments are shown (*n* = 3 cultures per group). **K** Chemotaxis of human CB CD34^+^ cells (left) and phenotypic HSC (right) migration toward human SDF-1, as quantified by flow cytometry. Data pooled from three independent experiments are shown (each dot represents an independent chemotaxis). (*n* = 6 cultures per group). **L** The percentage of human CD45^+^ cells in the BM of NSG mice 24 h after transplantation with 500,000 CB CD34^+^ cells that had been treated with vehicle or AGII (*n* = 3 mice per group). **M** The percentage of human CD45^+^ cells in the PB and BM of NSG mice at the indicated time points after transplantation with 10,000 CB CD34^+^ cells that had been treated with vehicle or AGII (*n* = 5 mice per group). **N** The percentage of human CD33^+^ myeloid cells and human CD19^+^ cells in the BM of NSG mice 4 months after transplantation with 10,000 CB CD34^+^ cells that had been treated with vehicle or AGII (*n* = 5 mice per group). **O**, **P** The frequency of human SRCs in CB CD34^+^ cells treated with vehicle or AGII, as determined by transplantations of graded doses of treated cells into NSG mice and determination of human CD45^+^ cell chimerism 4 months after transplantation (*n* = 30 mice in total). **Q** Human CD45^+^ cell chimerism in the BM of secondary recipient NSG mice at 4 months, which had been transplanted with 5 × 10^6^ BM cells from primary recipient NSG mice (*n* = 5 per group). Data shown as mean ± s.e.m. *p < 0.05; **p < 0.01; ***p < 0.001 by one-way ANOVA.

Overexpression of *FTO*, a m^6^A eraser, upregulates *CXCR4* expression in human CB CD34^+^ cells (Fig. 1A, B). Previous studies have shown that Angiotensin II (AGII) can activate *FTO* expression [26, 27]. Therefore, we assessed whether treatment of human CB CD34^+^ cell with AGII can active *FTO* expression. Using real-time PCR, we found after 20 h of treatment with AGII, *FTO* expression was significantly upregulated in human CB CD34^+^ cells (Fig. 6A). The binding between YTHDF2 and *CXCR4* was decreased, as well as the relative m6A labeled *CXCR4* level in human CB CD34^+^ cells (Fig. 6B, C). These results are consistent with the phenotypes of *FTO* overexpression in CB CD34+ cells (Fig. 1F, G). The results of flow cytometry analysis showed that AGII treatment upregulates the protein level of CXCR4 in human HSCs (Fig. 6D, E). To further confirm AGII upregulates CXCR4 expression through activation of *FTO* in human HSCs, we evaluated the effects of *FTO* inhibition on AGII treated human HSCs. Inhibition of *FTO* by meclofenamic acid or FB23-2 repressed the effects of AGII on CXCR4 upregulation (Fig. 6F) [28, 29]. By using shRNA, we found that downregulation of *FTO* greatly blocked AGII induced upregulation of CXCR4 in human HSCs (Fig. 6G–I). These results demonstrate that AGII upregulates CXCR4 expression in human HSCs through activation of *FTO*. In colony-forming assay, no significant difference was observed between AGII and vehicle-treated control group (Fig. 6J). AGII treatment also greatly enhanced human CB CD34^+^ cell migration toward graded doses of SDF-1, as well as chemotaxis to SDF-1 of HSCs (Fig. 6K). To evaluate whether enhanced migration of human CB CD34^+^ cells toward SDF-1 by AGII treatment depends on CXCR4 expression, we used AMD3100. AMD3100 treatment blocked the positive impact of AGII, demonstrating that AGII enhances the migration of human CB CD34^+^ cells via an active SDF-CXCR4 axis (Fig. 6K). In a homing assay, 20 h of CD34+ cell treatment with AGII dramatically promoted their homing to mouse BM (Fig. 6L). These results show that AGII can activate *FTO* expression in human CB CD34^+^ cells and eventually promote their migration and homing.

To assess the long-term reconstituting ability of AGII treated human CB CD34^+^ cells, we performed LDA to calculate the frequency of SRCs. AGII treated human CB CD34^+^ cells showed significantly increased engraftment in NSG recipients when compared to the control group, including increased human myeloid and lymphoid cell chimerism (Fig. 6M, N). The Poisson distribution analysis revealed the SRC frequency of 1/3984 for control human CB CD34^+^ cells and an enhanced SRC frequency of 1/1280 for AGII treated human CB CD34^+^ cells, indicating the presence of 251 and 781 SRCs in control and AGII treated cells, respectively (Fig. 6O, P). In secondary transplantation, the enhanced engraftment of AGII treated human CB CD34^+^ cells was apparent as compared to control group (Fig. 6Q). Thus, these results suggest that AGII treatment enhances human CB CD34^+^ cells engraftment.

## Discussion

Homing efficiency of HSCs is the key factor for successful HCT based clinical therapy. Although recent studies shed light on the biological functions of mRNA m^6^A modifications in HSCs, our work identifies *YTHDF2* and *FTO* as two important regulators of human CB CD34^+^ cell homing and engraftment. Repression of *YTHDF2* or activation of *FTO* in human CB CD34^+^ cells lead to upregulation of CXCR4 expression, thereby facilitating HSC migration, homing and engraftment. Using siRNA strategy or a library of compounds, we transiently repressed *YTHDF2* or activated *FTO* expression and show enhanced migration, homing and engraftment of human CB CD34^+^ cells. Our work provides multiple mechanisms and approaches to regulate CXCR4 expression in human CB CD34^+^ cells through inhibition of its mRNA decay, making its clinical application relatively convenient.

## Supplementary information

supplemental materials

## Data Availability

All data generated and/or analyzed during the current study are included in this published paper and its Supplementary Information.
