# Supplementary material for: Transient regulation of RNA methylation in human hematopoietic stem cells promotes their homing and engraftment
Source: Leukemia. 2022 Dec 2;37(2):453–64. doi: 10.1038/s41375-022-01761-4 (PMC9898034; doi:10.1038/s41375-022-01761-4)

Supplemental figure legends

Fig. S1 Downregulation of *YTHDF2* or upregulation of *FTO* inhibits CXCR4 decay. **A.** Representative tracks of *CXCR4* and *ATCB* harboring m6A peaks in human CB CD34+ cells. **B and C.** Schematic of the lentivirus used to knockdown or overexpress five m6a regulators. **D**. Histogram of surface CXCR4 protein level of sh-YTHDF1, sh-YTHDF2, sh-YTHDF3, OE-FTO or OE-ALKBH5 transfected human CB $CD34^+$ cells.

Fig. S2 Transient knockdown of *YTHDF2* by siRNA promotes human HSC migration and homing. A. Total YTHDF2 protein levels in Control or si-YTHDF2 transfected human CB $CD34^+$ cells as assessed by western blotting. A representative blot is shown. **B.** Representative FACS plots showed the phenotypic HSC ($CD34^+CD38^-CD45RA^-CD49f^+CD90^+$) population as assessed by flow cytometer. Gating was based on use of isotype control antibodies. The percentages indicate the frequencies of gated cell populations among the live cell events collected. **C.** Representative FACS plots showed the CXCR4 expression profile in HSCs ($CD34^+CD38^-CD45RA^-CD49f^+CD90^+$).

Fig. S3 Transient downregulation of *YTHDF2* by siRNA enhanced human CB $CD34^+$ cell engraftment. A, B. 10000 CB $CD34^+$ cells transfected with control or si-YTHDF2 were transplanted by i.v. injection into NSG mouse received 350 cGy total body irradiation one day beforehand. The percentage of human $CD33^+$ myeloid cell, $CD19^+$ B cell in bone marrow (BM) was determined 4 month after transplantation. (n=5

mice in control and si-YTHDF2 group). Representative pseudocolor plots are shown.

Fig. S4. A small-scale screen identified several compounds that functionally downregulate *YTHDF2* and enhance human CB CD34$^+$ cell migration and homing. **A.** Quantification of mean fluorescence intensity (MFI) of surface CXCR4 of human CB CD34$^+$ cells treated with Raloxifene for different time point. **B.** Quantification of mean fluorescence intensity (MFI) of surface CXCR4 of human CB CD34$^+$ cells treated with DMSO, different doses of Raloxifene. Representative data from two independent experiments are shown (n=5 cultures per group, one-way ANOVA). *P < 0.01; ***p<0.001 when compared with DMSO control. **C.** Quantification of mean fluorescence intensity (MFI) of surface CXCR4 of human CB CD34$^+$ cells treated with Imatinib for different time point. **B.** Quantification of mean fluorescence intensity (MFI) of surface CXCR4 of human CB CD34$^+$ cells treated with DMSO, different doses of Imatinib. Representative data from two independent experiments are shown (n=5 cultures per group, one-way ANOVA). *P < 0.01; ***p<0.001 when compared with DMSO control.

Supplemental Table 1.

primer name	5'-primer sequence-3'	
sh-YTHDF1-F	CACCCCCGAAAGAGTTTGAGTGGAACTCGAGTTCCACTCAAACTCTTTCGGG	shRNA Construction
sh-YTHDF1-R	AAAACCCGAAAGAGTTTGAGTGGAACTCGAGTTCCACTCAAACTCTTTCGGG	
sh-YTHDF2-F	CACCGCAGACTTGCAGTTTAAGTATCTCGAGATACTTAAACTGCAAGTCTGC	
sh-YTHDF2-R	AAAAGCAGACTTGCAGTTTAAGTATCTCGAGATACTTAAACTGCAAGTCTGC	
sh-YTHDF3-F	CACCGCAAGGAAATAAAGTTTCAGTCTCGAGACTGAAACTTTATTTCCTTGC	
sh-YTHDF3-R	AAAAGCAAGGAAATAAAGTTTCAGTCTCGAGACTGAAACTTTATTTCCTTGC	
sh-FTO-F1	CACCTCACGAATTGCCCGAACATTACTCGAGTAATGTTCGGGCAATTCGTGA	
sh-FTO-R1	AAAATCACGAATTGCCCGAACATTACTCGAGTAATGTTCGGGCAATTCGTGA	
sh-FTO-F2	CACCCCCATTAGGTGCCCATATTTACTCGAGTAAATATGGGCACCTAATGGG	
sh-FTO-R2	AAAACCCATTAGGTGCCCATATTTACTCGAGTAAATATGGGCACCTAATGGG	
GAPDH-F	TGTGGGCATCAATGGATTTGG	RT-qPCR
GAPDH-R	ACACCATGTATTCCGGGTCAAT	
ACTB-F	ACCGGGCATAGTGGTTGGA	
ACTB-R	ATGGTACACGGTTCTCAACATC	
Mir-145-F	GTCCAGTTTTCCCAGG	
Mir-145-R	GCGAGCACAGAATTAA	

Supplemental Table 2

Cell Condition	Cells transplanted	Number of mice with >1% human cell chimerism/total number of mice	SRC frequency
Control	1000<br>3000<br>10000	1/5<br>2/5<br>4/5	1/5824
si-YTHDF2	1000<br>3000<br>10000	3/5<br>5/5<br>5/5	1/894

Supplemental Figure 1

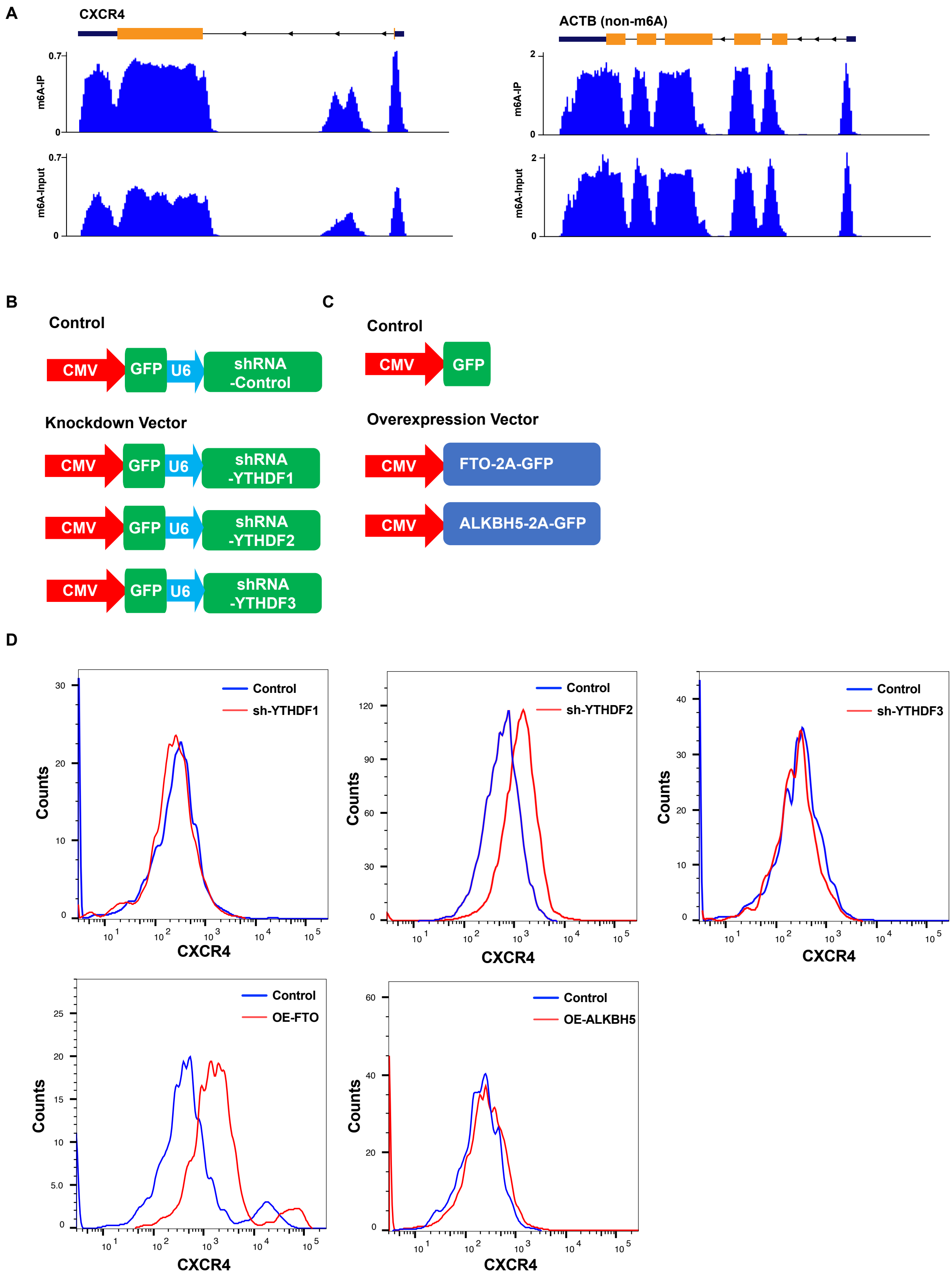

Supplemental Figure 2

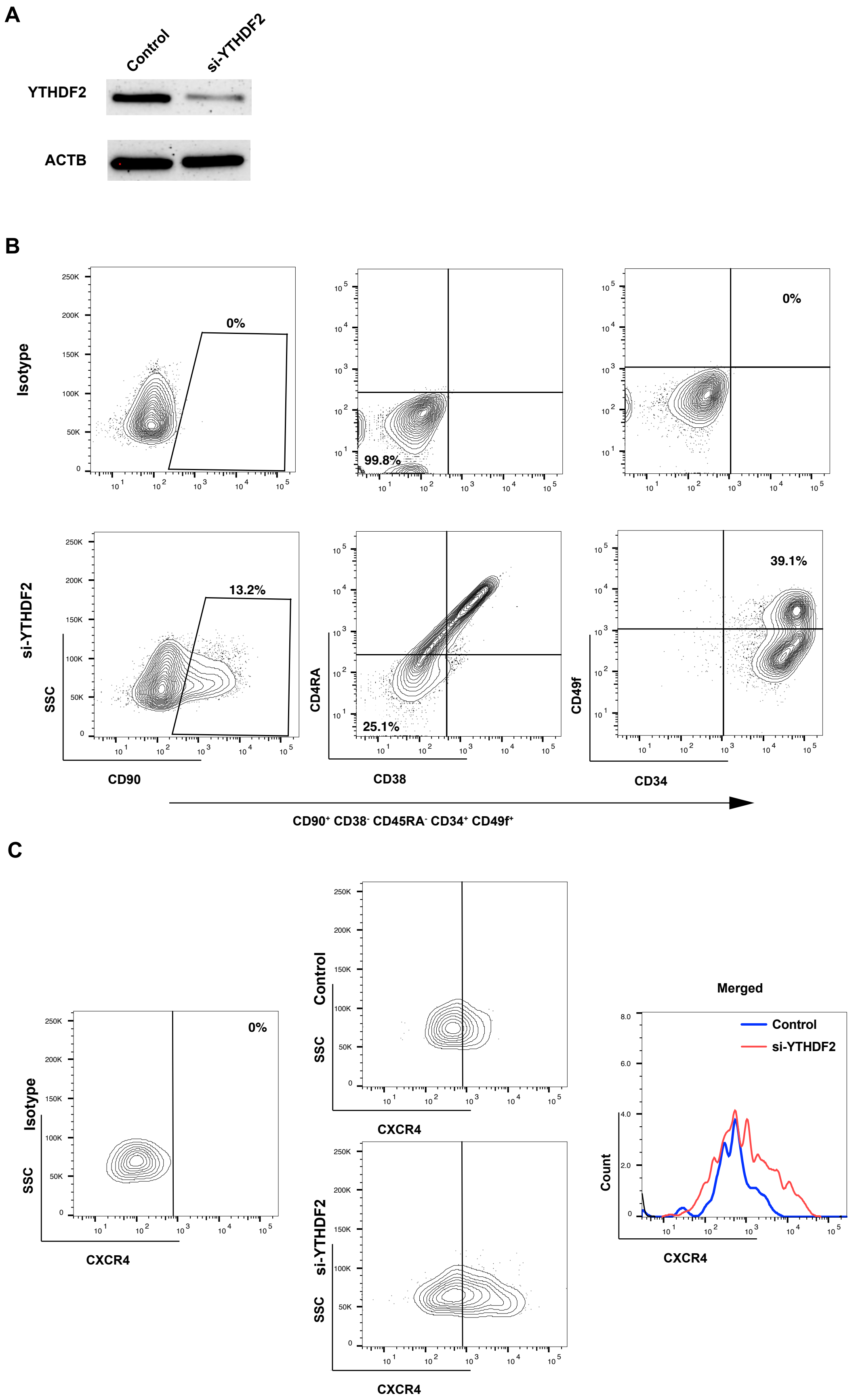

Supplemental Figure 3

A

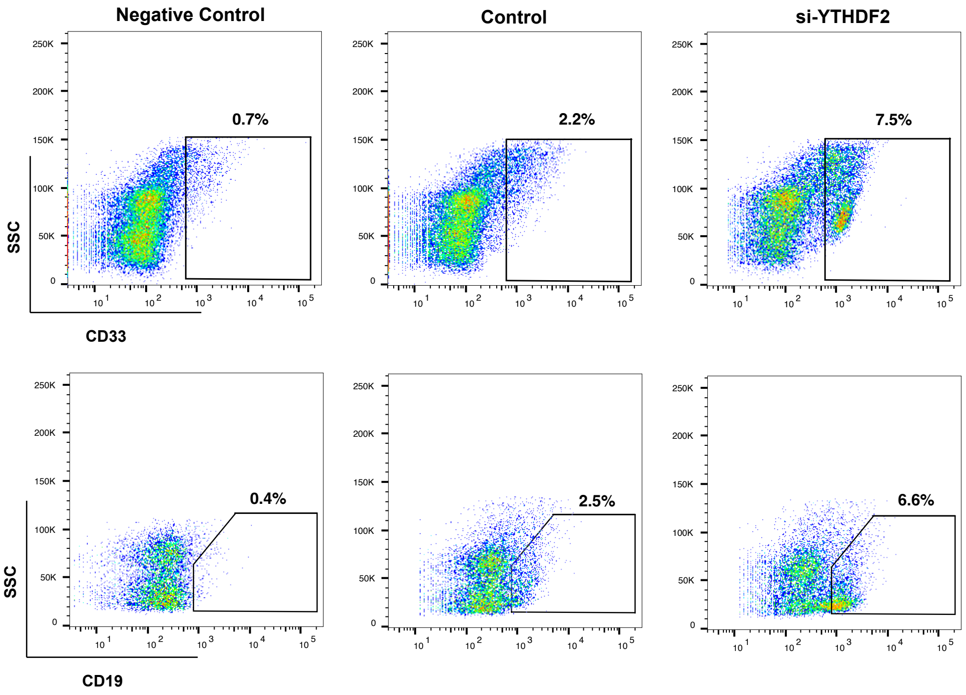

Supplemental Figure 4

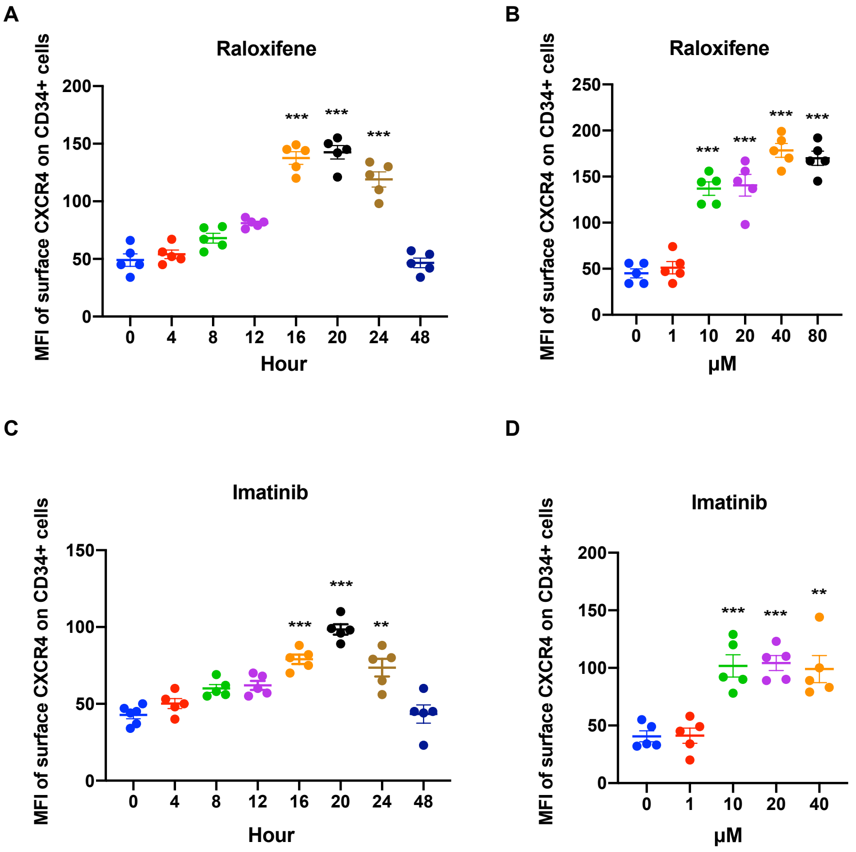

Supplement: Supplementary file 1 — supplemental materials [file 41375_2022_1761_MOESM1_ESM.pdf]
